# 175. Clindamycin versus linezolid for Group A Streptococcal bloodstream infections?: A comparative effectiveness study of adjunctive antitoxin therapy at 137 US Hospitals

**DOI:** 10.1093/ofid/ofad500.248

**Published:** 2023-11-27

**Authors:** Ahmed Babiker, Sarah Warner, Xioabai Li, Morgan Walker, Alexander Lawandi, Sameer S Kadri

**Affiliations:** Emory University School of Medicine, Atlanta, GA; Critical Care Medicine, National Institutes of Health Clinical Center, Bethesda, Maryland; National Institutes of Health, Bethesda, MD; 1. Critical Care Medicine Department, Clinical Center, National Institutes of Health, Bethesda, MD, 2. Critical Care Medicine Branch, National Heart Lung and Blood Institute, Bethesda, MD, Bethesda, Maryland; Department of Critical Care Medicine, National Institutes of Health Clinical Center, Bethesda, Maryland; National Institutes of Health Clinical Center, Bethesda, Maryland

## Abstract

**Background:**

Adjunctive clindamycin has survival benefit in invasive Group A streptococcus (GAS) infections. Like clindamycin, linezolid also leads to decreased toxin and virulence factor production. However, rising clindamycin resistance among GAS isolates and inadequate clinical data on linezolid both offer pause on which to choose. We examined the impact of adjunctive clindamycin vs. linezolid on survival among patients with GAS bloodstream infections (BSI) in the presence and absence of clindamycin resistant (clinda-R) isolates

**Methods:**

Clinical characteristics, antimicrobial susceptibility testing (AST), and antibiotic therapy were examined for unique adult inpatient encounters with GAS BSIs in the PINC-AI Database. Patients treated with a β-lactam for ≥3 days ±3 days of culture who received clindamycin ±3 days of culture were overlap weighted on a propensity-score to those who received linezolid using basine patient and hospital factors. The primary outcome was odds ratio (OR) of in-hospital mortality associated with clindamycin (vs linezolid). The secondary outcome was length of stay (LOS) among survivors. Subgroups analyses were conducted excluding clindamycin receipients with clinda-R isolates (subgroup 1) and also missing clindamycin AST(including D-test; subgroup 2).

**Figure 1.**
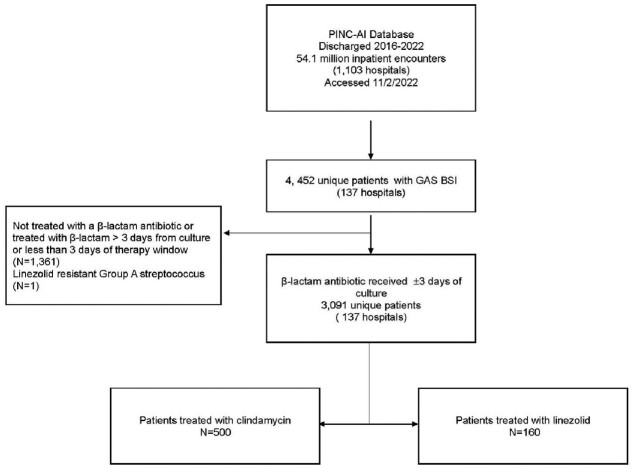
Study Flow chart

Selection of patients with Group A streptococcal blood stream infections. The database was queried for inpatients (aged ≥18 years) with blood culture displaying growth of Group A streptococcus, filtered on the basis of receiving β-lactam antibiotics within 3 days either side of culture sampling for a minimum duration of 3 days and received either adjunctive clindamycin or linezolid treatment within 3 days either side of culture sampling.

**Table 1:**
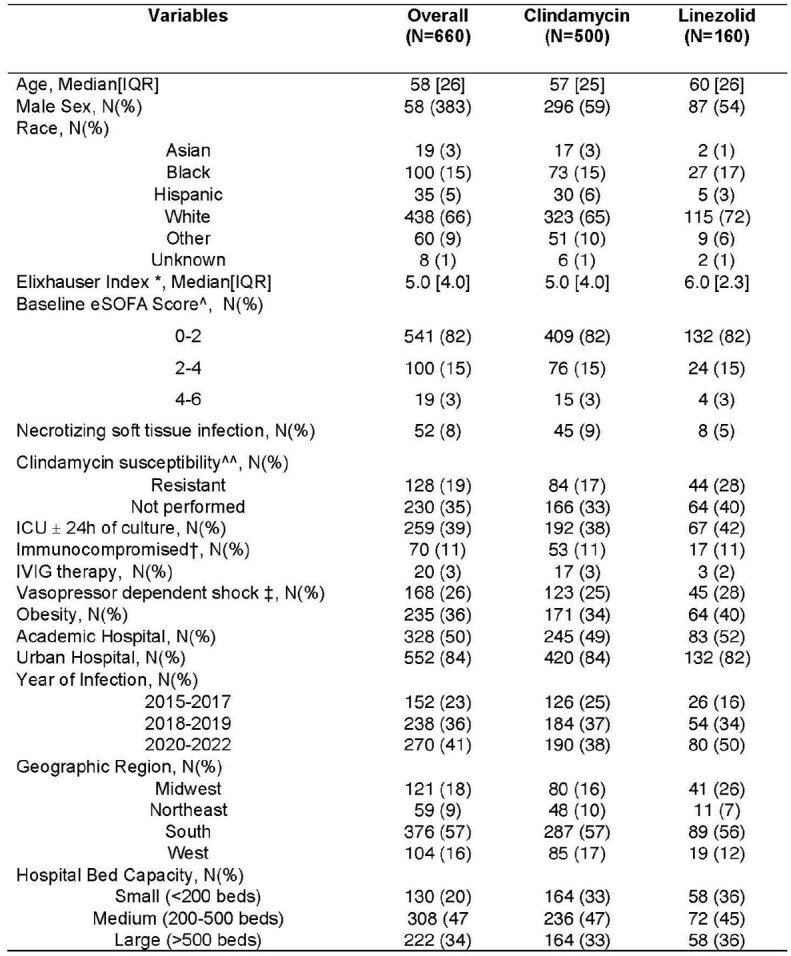
Baseline characteristics of all patients with Group A Streptococcal blood stream infections treated with β-lactam antibiotics and adjunctive clindamycin or linezolid (N=660) Abbreviations: IVIG: intravenous immunoglobulin, ICU: intensive care unit, IQR: interquartile range, SOFA: Sequential Organ Failure Assessment * This score was calculated by use of ICD-10-clinical modification codes and adapted from the methods used by Quan and colleagues ^ Calculated by use of an electronic health record-based adaption of the original Sequential Organ Failure Assessment ^^ Deteremined by clindamycin antimicrobial susceptibility and D-test results. Defined as resistant or intermediate clindamycin susceptibility results and/or a positive D-test result. † Defined as presence of ICD-10 codes coding for any of the following conditions: Human immunodeficiency virus, cancer, solid organ or hematopoietic stem cell transplantation, receipt of systemic corticosteroids, chemotherapy or other immunosuppressive therapy and immunodeficiency. ‡ Receipt of norepinephrine, epinephrine, phenylephrine, and dopamine administered within a 24-h period either side of culture sampling.

**Results:**

Of 3019 β-lactam–treated inpatients with GAS BSI, 500 (17%) received clindamycin and 160 (5%) received linezolid. The prevalence of clinda-R isolates was 19%;1 isolate was linezolid resistant and excluded (**Figure 1**). Overlap weighting resulted in well balanced groups (**Figure 2**). In the overlap weighted cohort, mortality risk was similar between recipients of clindamycin (10%, [50/500]) vs linezolid (9%, [14/160]; OR 1.38 [95% CI: 0.76-2.49]). Among survivors median[interquartile range] LOS was similar between the two groups (8[9] vs. 9[8] days, p=0.45). Removing those with clinda-R isolates (N=84) and also missing AST (N=166) yielded similar results (**Figure 3**).

**Figure 2.**
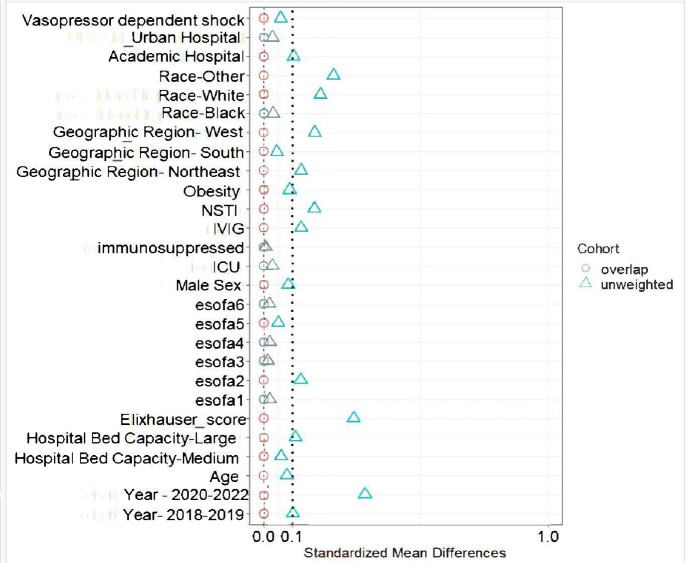
Standardized mean differences for covariates included the propensity score calculation

Standardized mean differences for covariates included in the propensity score generation averaged across exposure categories in the unweighted cohort (blue triangles) and overlap weighted cohort weight (red circles). After overlap weighting, the mean standard difference at each variable assessed was zero . Abbreviations: ICU: intensive care unit, IVIG: intravenous immunoglobulin, NSTI: necrotizing soft tissue infection

**Figure 3.**
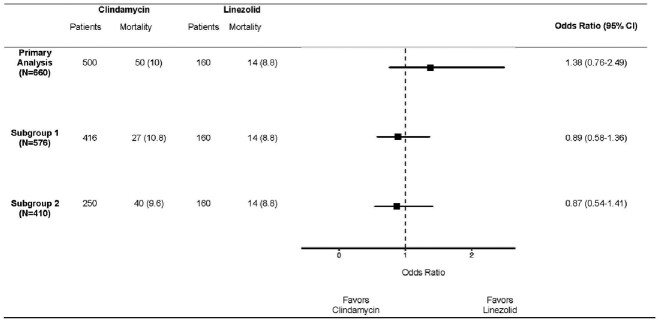
Odds Ratio of in-hospital mortality in patients with invasive group A streptococcal blood stream infection treated with adjunctive clindamycin versus adjunctive linezolid

The ORs (95% CIs) of in-hospital mortality (including discharge to hospice) in the primary analysis and subgroup analysis with patients withclindamycin resistant isolates (subgroup 1) and those with clindamycin resistant isolates and missing clindamycin susceptibility results (subgroup 2) removed from clindamycin group. Abbreviations: CI: confidence interval

**Conclusion:**

Among β-lactam-treated patients with GAS BSI, linezolid and clindamycin displayed comparable effectiveness as adjunctive antitoxin agents. Similar intrinsic effectiveness (i.e., in patients with only susceptible isolates) supports linezolid as an alternative even in low clinda-R settings.

**Disclosures:**

**Ahmed Babiker, MBBS**, Roche: Advisor/Consultant **Morgan Walker, MD**, Cytovale: Advisor/Consultant

